# Effect of maternal Tdap on infant antibody response to a primary vaccination series with whole cell pertussis vaccine in São Paulo, Brazil

**DOI:** 10.1016/j.jvacx.2021.100087

**Published:** 2021-02-16

**Authors:** Lourdes R.A. Vaz-de-Lima, Ana Paula S. Sato, Lucia C. Pawloski, Eder G. Fernandes, Gowrisankar Rajam, Helena K. Sato, Divya Patel, Han Li, Euclides A. de Castilho, Maria Lucia Tondella, Jarad Schiffer, on behalf of the Maternal Pertussis Vaccine Working Group

**Affiliations:** a Centro de Imunologia, Instituto Adolfo Lutz, Coordenadoria de Controle de Doenças da Secretaria de Estado da Saúde, São Paulo, Brazil; b Departmento de Epidemiologia, Faculdade de Saúde Pública, Universidade de São Paulo – USP, Brazil; c Division of Bacterial Diseases, NCIRD, Centers for Disease Control and Prevention, Atlanta, GA, USA; d Divisão de Imunização, Centro de Vigilância Epidemiológica Prof. Alexandre Vranjac, Coordenadoria de Controle de Doenças da Secretaria de Estado da Saúde SP, Brazil; e Faculdade de Medicina –USP, Brazil

**Keywords:** Pertussis, Maternal antibodies, Tdap, Infant vaccination, DTwP, Blunting

## Abstract

**Background:**

Maternal Tetanus, diphtheria, and acellular pertussis (Tdap) vaccination provides antibody transfer to newborn infants and may affect their antibody response to the primary vaccination series. This study aimed to assess the effect of Tdap vaccination during pregnancy on infant antibody response to the whole cell pertussis (DTwP) primary series.

**Methods:**

Plasma from 318 pregnant women (243 Tdap-vaccinated and 75 unvaccinated) and their infants (cord blood) was collected at delivery; infant blood was again collected at 2 and 7 months, before and after their primary DTwP series. Anti-pertussis toxin (PT), pertactin (PRN), filamentous hemagglutinin (FHA), fimbriae 2/3 (FIM) and adenylate cyclase toxin (ACT) IgG antibodies were quantified by a microsphere-based multiplex antibody capture assay and anti-PT neutralizing antibodies by the Real Time Cell analysis system.

**Results:**

Infant geometric mean concentrations (GMCs) of IgG anti-Tdap antigens were significantly higher (p < 0.001) among the Tdap-vaccinated (PT: 57.22 IU/mL; PRN: 464.86 IU/mL; FHA: 424.0 IU/mL), versus the unvaccinated group (4 IU/mL, 15.43 IU/mL, 31.99 IU/mL, respectively) at delivery. Anti-FIM and ACT GMCs were similar between the two groups. At 2 months of age, anti-PT, PRN, and FHA GMCs remained higher (p < 0.001) in the Tdap-vaccinated group (12.64 IU/mL; 108.76 IU/mL; 87.41 IU/mL, respectively) than the unvaccinated group (1.02 IU/mL; 4.46 IU/mL; 6.89 IU/mL). However, at 7 months, after receiving the third DTwP dose, the anti-PT GMC was higher (p = 0.016) in the unvaccinated group (7.91 IU/mL) compared to the vaccinated group (2.27 IU/mL), but without differences for anti-PRN, FHA, FIM and ACT GMCs.

**Conclusion:**

Elevated antibody levels suggest that maternal Tdap vaccination might protect infants until 2 months of age. Reduced anti-PT levels at 7 months indicate potential blunting of immune response in infants. Surveillance would help determine if blunting alters vaccine immunity and impacts pertussis prevention in infants.

## Highlights

• High antibody levels obtained from maternal Tdap might protect infants until 2 months.
• Reduced anti-PT IgG at 7 months of age indicate potential blunting of immune response.
• Surveillance of infants would help determine if blunting alters vaccine immunity.

## 1 Introduction

Pertussis continues to be one of the most serious bacterial diseases of public health relevance, despite prevention efforts, with several gaps in our understanding of the immunology and pathogenesis of this disease. Considered a reemerging disease, the incidence of pertussis has increased in several countries, including Brazil [1], [2], [3]. The most vulnerable groups are neonates and young infants (mainly those aged <2 months) without vaccination in whom severe morbidity (hospitalization) and higher pertussis-associated mortality exists [4], [5], [6].

Pertussis maternal vaccination during pregnancy has been employed as a strategy to protect the infants throughout the world [7], [8], [9], [10], [11]. Maternal vaccination has shown promising results in demonstrating protection of infants in several countries [8], [12], [13], [14].

Maternal vaccination with acellular pertussis vaccine was introduced in November 2014 in Brazil and was recommended at first from 27 to 36 weeks of pregnancy [15] and subsequently changed in 2017 to starting at the 20th week [16]. The maternal placental transfer of pertussis-specific antibodies to infants can protect them in the first months of life. However, it may affect their own immune response later, when they receive their primary pertussis vaccination series. Blunting, an inhibition of antibody response, has been previously observed [17], [18], [19], [20], [21], [22], [23], [24], [25], [26] but its clinical relevance is still unknown [13], [27], [28].

Brazil’s current pertussis immunization program consists of a pentavalent vaccine that contains 3 doses of the diphtheria-tetanus-whole-cell pertussis (DTwP) + Haemophilus influenzae b + hepatitis B (DTwP-Hib-HBV) administered at 2, 4 and 6 months of age, followed by two boosters doses of DTwP at 15 months and 4 years [29]. To the best of our knowledge, there are few studies conducted on the effect of maternal vaccination on antibody response to the routine vaccination in infants receiving whole cell pertussis vaccines [30], [31].

Despite the lack of a correlate of protection for pertussis, high levels of IgG antibodies to pertussis toxin (PT), pertactin (PRN) and filamentous hemagglutinin (FHA) have been suggested as indicative of protection against pertussis [32], [33], [34]. The aim of the present study was to assess the effect of tetanus, diphtheria, and acellular pertussis (Tdap) vaccination during pregnancy on the infant antibody response to the DTwP primary series.

## 2 Methods

### 2.1 Study population and procedures

In this prospective cohort study, a total of 318 pregnant women (243 Tdap-vaccinated and 75 unvaccinated) and their infants were enrolled from July 2015 to March 2017, in São Paulo, Brazil. The recruitment was by convenience and took place during their delivery hospitalization from two different Maternity hospitals: *Hospital and Maternity Interlagos* and the *Hospital Leonor Mendes de Barros.*

The exclusion and inclusion criteria and questionnaire with demographic and socioeconomic data from all the pregnant women who accepted to participate were previously described [35]. An additional questionnaire on growth parameters, breastfeeding, hospitalization and day care attendance was completed at 2 and 7 months on the infants.

Sample size was estimated based on the geometric mean concentration (GMC) difference of 20 International Units (IU)/mL (standard deviation of 30 IU/mL for each group) of anti-PT IgG of the cord blood among the vaccinated group compared to the unvaccinated group at delivery, confidence interval (two-sided) of 95%, and test power of 80% (minimum of 36 individuals for each group, vaccinated and unvaccinated).

The *Instituto Adolfo Lutz* Ethics Committee approved the study and participants gave their written informed consent. This study was also reviewed in accordance with CDC human research protection procedures and CDC was determined to be non-engaged in human subjects research; CDC IRB approval was therefore not required.

Vaccination status of mothers and infants were verified by vaccination records and confirmed with the centralized Information System from National Immunization Program (SI-PNI). In this study, all infants received three doses of DTwP vaccine at 2, 4 and 6 months.

Maternal plasma samples were collected within 24 h of delivery; infant blood samples were collected at birth (cord blood), 2 months of age (before vaccination), and 7 months of age (about 1 month after the third vaccine dose of primary DTwP series). Maternal, cord blood and infant samples were centrifuged to collect plasma at the hospitals within 24 h after blood collection. Plasma samples were aliquoted, coded and stored at −80 °C at the Laboratory of Pertussis Serology of the *Instituto Adolfo Lutz*. Aliquots of each plasma sample were shipped on dry ice to the Microbial Pathogenesis and Immune Response Laboratory (MPIR), CDC, Atlanta, Georgia, USA, for testing.

### 2.2 Maternal Tdap and DTwP in infants

The vaccine used for all pregnant women was Boostrix® (GSK Biologicals, Rixensart, Belgium), licensed in Brazil as Refortrix®, which contains 8 μg of inactivated pertussis toxoid, 8 μg of FHA, and 2.5 μg of PRN, 20 International Units (IU) of tetanus toxoid (TT), and 2 IU of diphtheria toxoid (DT).

All infants were vaccinated with the DTwP-Hib-HBV Conjugate vaccine-Pentavalent Vaccine (Serum Institute of India Ltd., Pune, India), containing ≥40 IU of TT, ≥30 IU of DT, ≥4 IU of wP, ≥10 μg of HBsAg, and 10 μg of purified capsular Hib polysaccharide (PRP) conjugated to TT (carrier protein) adsorbed on aluminium phosphate, Al ≤ 1.25 mg.

### 2.3 Laboratory methods

#### 2.3.1 Microsphere-based multiplex antibody capture assay (MMACA)

Antibodies IgG against PT, PRN, FHA, fimbriae (FIM) and adenylate cyclase toxin (ACT) were quantified by a MMACA as per the standard operating procedures provided by the MPIR Laboratory, CDC [36].

Briefly, all samples were diluted in a 96-well round bottom titer plate (CLS3799, Sigma, St Louis, MO, USA) with the assay buffer (PBS-2.5%BSA-0.05% azide, pH 7.4). The plasma samples were diluted 2-fold for 7 dilutions starting at 1/50. Each assay plate included a pertussis human standard (WHO International Standard, 06/140, NIBSC; UK) diluted in duplicate 4-fold for 8 dilutions, starting at 1/20, and internal quality controls (QC): positive control (WHO Reference Reagent, 06/142, NIBSC; UK) diluted in duplicate 2-fold for 4 dilutions starting at 1/400; negative control (IgG–free human serum, Sigma, St. Louis, MO, USA) in duplicate, and assay buffer control (blank). This was incubated with 25 µL microspheres conjugated to pertussis antigen (multiplex, 2500 microspheres/protein/well) and R-Phycoerythrin (R-PE) goat anti-human Fcγ specific IgG (Moss Inc., Pasadena, MD, USA) was used as the reporter antibody. The plate was read in a Luminex 200 plate reader (Luminex Corp., Houston, TX, USA). The mean fluorescence intensity (MFI) of the reporter antibody is directly proportional to the amount of antigen-specific antibody bound to a given microsphere set. Data was analyzed with SAS program version 9.3 (SAS Institute Inc., Cary, NC, USA) running a MMACA customized endpoint algorithm to measure the anti-pertussis antigen specific antibody concentration. The lower limit of quantitation (LLOQ) of the assay was 0.08 IU/mL for PT, 0.04 IU/ml for PRN, 0.15 IU/mL for FHA, 0.06 IU/mL for FIM and 0.09 IU/mL for ACT [36]. Paired mother and infant samples were blinded and tested on the same plate.

#### 2.3.2 Toxin neutralization assay (TNA)

TNA was performed to assess the functionality of anti-PT antibodies before and after the primary DTwP series in infant plasma samples. A representative sampling was performed of the larger population of samples. Infant samples were divided into six groups, stratified by timing of blood collection and status of maternal vaccination (Tdap-vaccinated or unvaccinated mother): cord blood/vaccinated, cord blood/unvaccinated, 2 months/vaccinated, 2 months/unvaccinated, 7 months/vaccinated, and 7 months/unvaccinated. Within each of the six groups, samples were sorted by highest to lowest MMACA IgG anti-PT levels; every other sample was then selected for testing (total = 309).

Briefly, samples were serially diluted across a 96-well tissue culture plate from 50-fold to 3200-fold and pre-incubated with active PT. This mixture of the plasma and PT was incubated for 30 min at 37 °C in 5% CO_2_, and added to Chinese hamster ovary-K1 cells (ATCC Cat# CCL-61) and incubated again at 37 °C, followed by an assessment of morphological alterations using the xCELLigence™ Real Time Cell analysis system (RTCA, Acea Biosciences, Inc.), read as a cell index [37]. The timepoint where the toxin had maximum impact on the control cells was chosen for analysis (usually ~10–11 h post-intoxication). The cell index of each dilution was fit to a 4-PL curve, and the midpoint was identified as the Effective Dilution 50% (ED50), the point at which 50% of the toxin effect was neutralized. The ED50 was then normalized against the ED50 of the WHO 06/140 reference sera which was run as a reference standard on each plate. Geometric mean titers (GMTs) of ED50 were calculated in each group based on individual ED50 values for group comparison.

### 2.4 Statistical analysis

Descriptive statistics consisted of absolute and relative frequencies and means and standard deviations of characteristics of mothers and infants. These variables were compared between vaccinated and unvaccinated groups, using the Chi-squared test, with a 5% significance level.

GMCs with 95% confidence intervals (95% CI) of maternal and infant antibodies were calculated. Comparison of GMCs between groups (vaccinated and unvaccinated) was done using the Student’s *t*-test for data that was normally distributed or Mann Whitney test. Statistical analyses were performed using Stata 12 Software (StataCorp LLC, Texas, USA) and GraphPad Prism Software 5 (GraphPad Software, CA, USA).

## 3 Results

### 3.1 Study population

**Fig. 1:**
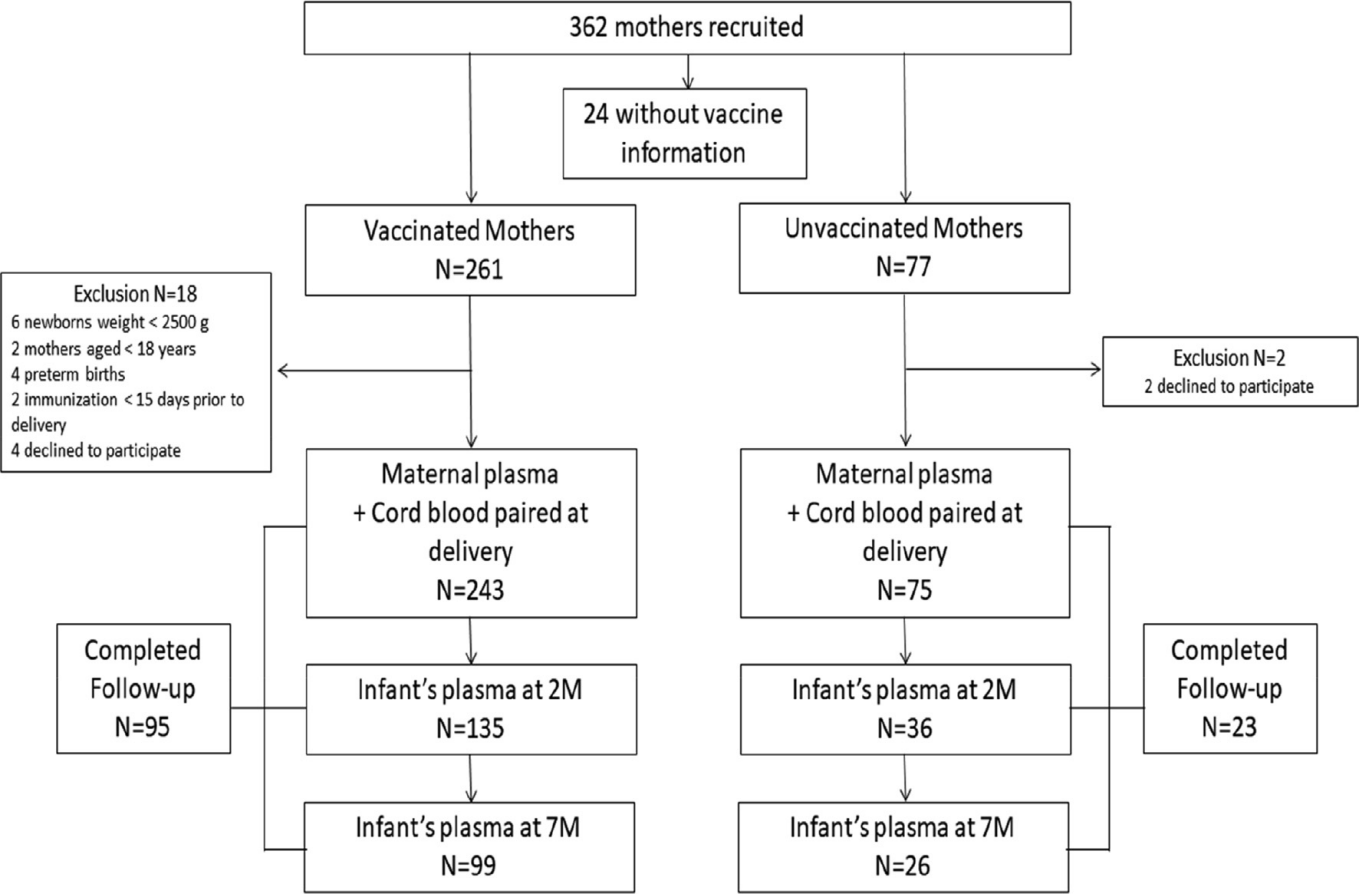
Flow chart of study. Abbreviations: M = months.

A total of 318 pregnant women and their infants were enrolled in this study. Fig. 1 shows the flow chart of the study. Of the total enrolled, 95 infants of the 243 (39.1%) vaccinated mothers completed the follow-up blood collection schedule at 2 and 7 months; 23 infants of the 75 (30.7%) unvaccinated mothers completed their follow-up collection. We observed that those who missed the follow-up do not differ from those who remained in the cohort, apart from two variables (maternal race and birthweight) from the eight socio-demographic, pre-natal and delivery characteristics from mothers and their infants (Supplementary Table S1). Supplementary Table S2 shows the characteristics of infants included in this study. Regarding breastfeeding, most children were breastfeeding at 2 (95.3%) and 7 months (64.5%), and most of them did not attend day care (87.1%).

### 3.2 Maternal antibody response at delivery

As shown in Table 1, maternal GMCs of the anti-Tdap vaccine antigens (PT, PRN, FHA) were significantly higher among the vaccinated group compared to the unvaccinated group (p < 0.001) at delivery. On the other hand, GMCs of anti-FIM and anti-ACT (which are not in the maternal vaccine) were similar in both groups.

**Table 1:** Geometric mean concentration (GMC) of IgG antibodies against pertussis antigens PT, PRN, FHA, FIM, and ACT, of vaccinated and unvaccinated mothers at delivery.

		**Vaccinated**			**Unvaccinated**		
	**N**	**GMC**	**95% CI**	**N**	**GMC**	**95% CI**	p*
**PT**	243	43.51	37.51–50.47	75	4.69	3.23–6.80	<0.001
**PRN**	243	406.17	329.33–500.94	75	15.67	10.70–22.93	<0.001
**FHA**	243	314.10	278.74–353.93	75	41.64	31.34–55.32	<0.001
**FIM**	243	19.06	15.61–23.28	75	15.7	10.84–22.74	0.378
**ACT**	232	41.95	37.42–47.04	71	41.64	32.50–53.34	0.545

* Mann-Whitney test.

### 3.3 Influence of maternal Tdap vaccination on the infant antibody response

**Fig. 2:**
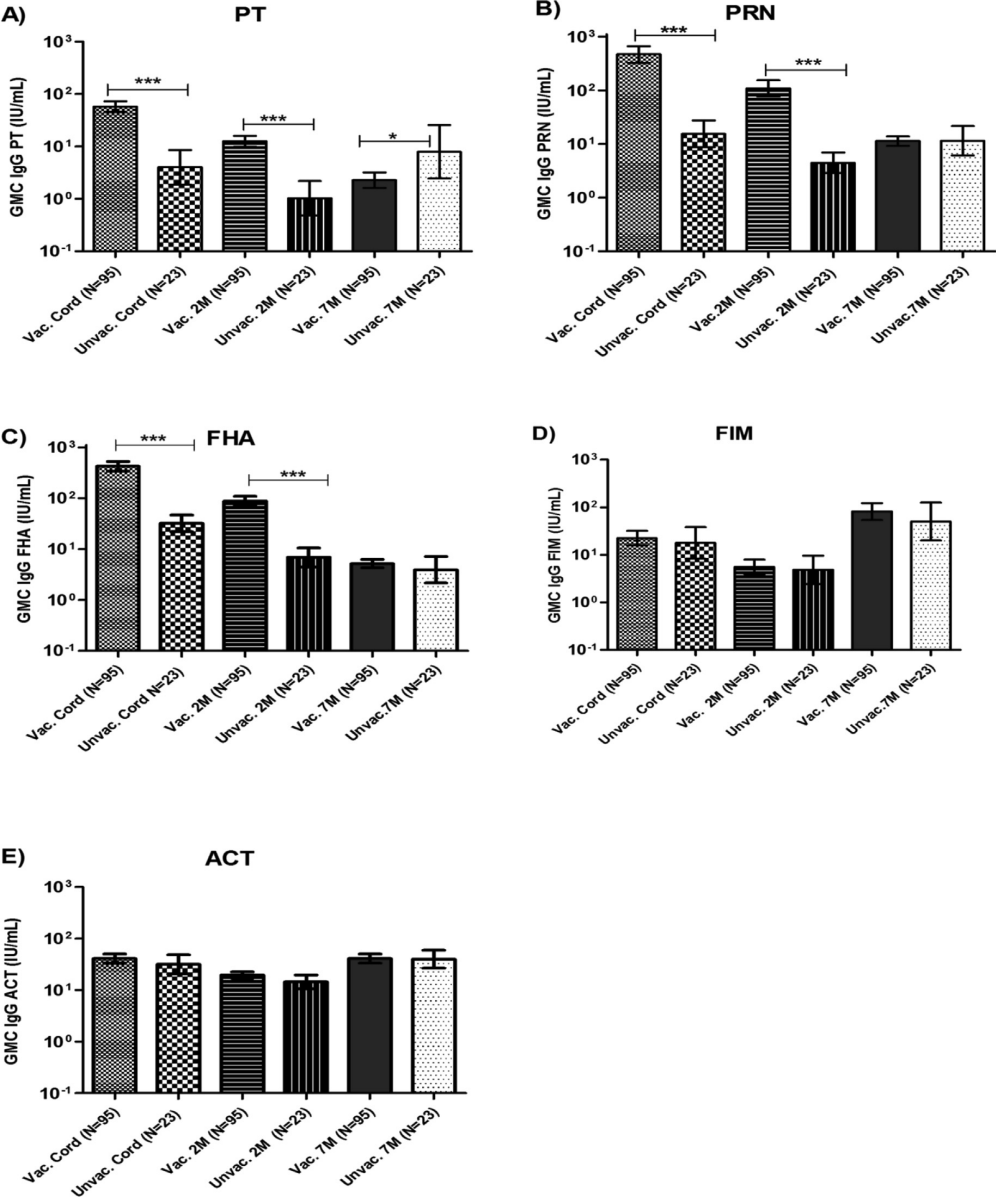
Geometric mean concentrations (GMCs) for IgG antibodies to pertussis antigens PT (A), PRN (B), FHA (C), FIM (D) and ACT (E) in cord blood at delivery, infant plasma before primary vaccination (2M) and 1 month after the third vaccine dose (7M) for only those mother-infant pairs that finished complete follow-up. Statistical significance is indicated *= P < 0.05; ***= P < 0.001. Mann-Whitney test was used to analyze all variables, except for FHA, PRN and ACT at 7 months (Student’s *t* test). The number of samples tested (N) for time points was indicated in graphs.

Fig. 2 shows the GMCs for IgG antibodies against pertussis antigens PT, PRN, FHA, FIM and ACT in infant plasma at birth (cord blood), before primary DTwP vaccination at 2 months (2 M) and 1 month after the third vaccine dose at 6 months (7 M) for only the infants that completed the follow-up collection from delivery until 7 months. At delivery, infant GMCs of antibodies to the Tdap vaccine antigens (PT, PRN, FHA) were significantly higher (p < 0.001) among the maternal Tdap-vaccinated group (anti-PT: 57.22 IU/mL, 95%CI 44.79–73.11; anti-PRN: 464.86 IU/mL, 95%CI 324.84–665.21; and anti-FHA: 424.0 IU/mL, 95%CI 340.69–527.70) compared to the unvaccinated group (anti-PT: 4 IU/mL, 95%CI 1.87–8.54; anti-PRN: 15.43 IU/mL, 95%CI 8.66–27.51; and anti-FHA: 31.99 IU/mL, 95%CI 21.98–46.53). Both GMCs of anti-FIM and anti-ACT antibodies were similar between the maternal Tdap-vaccinated (anti-FIM: 22.48 IU/mL, 95%CI 15.77–32.03; and anti-ACT: 40.84 IU/mL, 95%CI 33.27–50.13) and unvaccinated groups (anti-FIM: 17.77 IU/mL, 95%CI 8.22–38.42; and anti-ACT: 31.56 IU/mL, 95%CI 20.57–48.42).

At 2 months of age, anti-PT, anti-PRN, and anti-FHA antibody concentrations declined but remained higher (p < 0.001) in the maternal Tdap-vaccinated group (anti-PT: 12.64 IU/mL, 95%CI 9.95–16.05; anti-PRN: 108.76 IU/mL, 95%CI 76.72–154.17; and anti-FHA: 87.41 IU/mL, 95%CI 70.51–108.36) compared to the unvaccinated group (anti-PT: 1.02 IU/mL, 95%CI 0.47–2.19; anti-PRN: 4.46 IU/mL, 95%CI 2.85–6.96; and anti-FHA: 6.90 IU/mL, 95%CI 4.49–10.56). In this age group, anti-FIM and anti-ACT GMCs were similar between the maternal Tdap-vaccinated (anti-FIM: 5.56 IU/mL, 95%CI 3.89–7.95; and anti-ACT: 19.58 IU/mL, 95%CI 16.87–22.72) and unvaccinated (anti-FIM: 4.82 IU/mL, 95%CI 2.41–9.65; and anti-ACT: 14.45 IU/mL, 95%CI 10.71–19.51).

However, at 7 months, after receiving the third DTwP dose, the anti-PT GMC was higher (p = 0.016) in the unvaccinated group (7.91 IU/mL; 95%CI 2.43–25.68) compared to the maternal Tdap-vaccinated group (2.27 IU/mL; 95%CI 1.61–3.20), with no differences in the anti-PRN, anti-FHA, anti-FIM and anti-ACT GMCs in both groups.

Supplementary Figure S1 shows the GMCs for IgG antibodies against pertussis antigens PT, PRN, FHA, FIM and ACT in infant plasma at birth (cord blood), before primary DTwP vaccination (2 M) and 1 month after the third vaccine dose (7 M) for all infant samples collected. Results of this group were similar to results in Fig. 2 at all timepoints, including 7 months, where the anti-PT GMC was higher (p = 0.050) in the unvaccinated group (6.32 IU/mL, 95%CI 2.17–18.37) compared to the maternal Tdap-vaccinated group (2.28 IU/mL, 95%CI 1.61–3.19), with no differences in the anti-PRN, anti-FHA, anti-FIM and anti-ACT GMCs in both groups.

### 3.4 Breastfeeding and infant IgG response

Supplementary Table S3 shows the anti-PT, PRN, FHA, FIM and ACT GMCs in infants at 2 months, stratified by maternal vaccine status and breastfeeding. Breastfeeding seems to positively influence the anti-PT, PRN, and FHA levels of infants whose mothers were vaccinated; however, the difference was only significant for anti-FHA (p = 0.029), likely due to low infant numbers in the non-breastfeeding groups. By 7 months of age, this difference is no longer observed (Supplementary Table S4).

### 3.5 Anti-PT neutralizing antibodies

Functional antibody level was assessed by measuring the anti-PT neutralizing antibodies in a subset of plasma samples. As observed in Table 2, GMTs of anti-PT antibodies in cord blood at delivery and in infant plasma before primary vaccination (2 M) were significantly higher among the infants born to vaccinated mothers compared to those born to the unvaccinated ones (p < 0.001). Nevertheless, infants born to vaccinated mothers had lower GMT anti-PT IgG antibodies at 7 months, after receiving the third dose of the DTwP vaccine (around one month after the primary vaccination series) compared to the infants born to the unvaccinated group. GMT of ED50 was 63.86 in the vaccinated group versus 95 in the unvaccinated group, but the difference was not statistically significant (p = 0.231).

**Table 2:** Geometric mean titers (GMTs) of infant IgG anti-PT neutralizing antibodies at delivery (cord blood), before primary vaccination (2 month, 2M), and 1 month after the third vaccine dose (7 month, 7M) in vaccinated and unvaccinated groups.

	**Vaccinated**			**Unvaccinated**			
	N	GMT	95% CI	N	GMT	95% CI	p*
**Cord**	128	234.73	203.84–270.29	32	61.53	45.44–83.31	<0.001
**2M**	68	112.32	95.99–131.41	18	61.27	45.88–81.82	<0.001
**7M**	50	63.86	52.37–77.86	13	95.5	49.56–184.04	0.231

* Mann-Whitney test.

## 4 Discussion

To our knowledge, there are few studies of maternal antibodies interference to infants’ immunization with DTwP in middle income countries [38]. That includes a study carried out in Thailand [31] and ours conducted in Brazil.

In this study, maternal and infant GMCs of all the Tdap vaccine antigens (PT, PRN and FHA) were significantly higher among the vaccinated group compared to the unvaccinated group (p < 0.001) at delivery. At 2 months of age, GMCs of anti-PT, anti-PRN, and anti-FHA remained higher in the Tdap-vaccinated group (p < 0.001), suggesting that these antibodies might be contributing to the protection against pertussis on newborn infants in this period of higher morbidity and mortality. Functional toxin-neutralizing antibodies were also found in infants from the vaccinated group at delivery and 2 months, providing further evidence of the protective antibodies that are likely getting transferred during pregnancy. Among the subject-specific variables, we found that breastfeeding may be associated with higher FHA antibodies in infants for the first 2 months of age whose mothers were vaccinated, but this difference is no longer observed by 7 months.

However, at 7 months of age, after receiving the third DTwP dose, the anti-PT IgG GMC was higher in the unvaccinated group (p < 0.05), despite a borderline statistically significant difference that should be interpreted with caution, suggesting the possibility of a blunting effect of the circulating maternal antibodies on infant vaccine immune response. A similar trend was observed with PT-neutralizing antibodies (Table 2) but this was not statistically significant likely due to the small sample size.

This observation is consistent with those demonstrated by Englund et al. and Wanlapakon et al. [30], [31] in infants who received 3 doses of DTwP using the same schedule of 2, 4 and 6 months of age, although in the former study, the women were not vaccinated during pregnancy and in the latter a blunting effect was observed for anti-FHA IgG response. Other infant vaccine response studies that also found blunting for IgG antibodies against PT and other vaccine antigens after the primary DTaP series [21], [39] of 2, 4 and 6 months showed that the blunting effect disappeared after the booster vaccine [17], [20]. While the basic mechanism of immune blunting still remains unclear, several hypotheses are being actively deliberated and scientifically challenged in the research community [40].

In our study, a low PT response was observed for both groups, including the infants whose mothers did not receive vaccination. To our knowledge, there are very few studies that investigated the anti-PT response of DTwP in Brazilian infants [41], [42], [43] and only one, Zorzeto *et al.*, measured anti-PT GMC antibodies. They found levels of DtwP with low Lipopolysaccharide (LPS) content compared with the conventional DTwP were 12.65 IU/mL and 14.07 IU/mL, respectively, which is higher than our results for the group without maternal vaccination (7.9 IU/mL). However, such comparisons can be complex due to the difference in vaccine manufacturers, the different bacterial strains used to produce the vaccines [44], and even the different methodologies used to measure the immune response [20]. It is also worth pointing out that the DTwP vaccines usually used in the Brazilian National Immunization Program are produced by the Butantan Institute São Paulo, Brazil, but during this study, due to the lack of this vaccine, a DTwP vaccine from Serum Institute of India was used instead.

In this study, we found a blunting effect only for PT. PT is the main pertussis antigen related to protection [45], [46], as shown by Kapil et al. [47], who found with their baboon model that PT alone was enough to protect the offspring from pertussis disease. However, anti-FHA, anti-PRN and anti-FIM antibodies have also been associated with protection by preventing pathogen adherence to the host epithelium [48]. Studies from Guiso et al and Sebo et al showed that ACT is also an important antigen with protective activity [49], [50]. Regardless, it is important to consider that although blunting was observed for PT, the clinical relevance of this observed blunting effect is unknown, and no evidence has been found that blunting is associated with increased risk of disease. Yet it is relevant to note that these data have been shown in studies conducted in high income countries, such as the UK and US [13], [27]. The clinical relevance of the blunting effect of the antibody response in infants vaccinated with DTwP born to vaccinated mothers is more difficult to evaluate in low-middle income countries than infants born to vaccinated mothers in high-income countries due to differences in the pertussis surveillance policies, subject compliance and logistics [38]. Furthermore, despite blunting for PT, infants should still be receiving protective immunity from many more antigens within the whole cell pertussis vaccine.

This study has limitations such as the decreased number of participants for the follow-up at 7 months of age. The losses for the follow-up should not be seen as a severe study limitation because we observed no statistically significant difference in six sociodemographic and clinical characteristics between those who missed the follow-up and those who remained in the cohort, which suggests there is no bias selection.

Secondly, this study was conducted at public hospitals located in two different regions of the Sao Paulo and may not be necessarily representative of the total population of the city. Finally, pre-vaccination maternal plasma could not be obtained to analyze the effect of maternal Tdap vaccination on the mothers’ antibody levels.

In conclusion, elevated anti-pertussis antigen specific antibody levels in infants suggest maternal Tdap vaccination might protect infants during the first 2 months of age. Reduced anti-PT levels in the Tdap-vaccinated group at 7 months of age indicate a potential blunting of antibody response. Surveillance of infants at this age could help determine if blunted antibody response alters vaccine immunity and impacts pertussis prevention and control in this age group.

## Declaration of Competing Interest

The authors declare that they have no known competing financial interests or personal relationships that could have appeared to influence the work reported in this paper.
