# Supplementary material for: Effect of maternal Tdap on infant antibody response to a primary vaccination series with whole cell pertussis vaccine in São Paulo, Brazil
Source: Vaccine X. 2021 Feb 16;7:100087. doi: 10.1016/j.jvacx.2021.100087 (PMC8010450; doi:10.1016/j.jvacx.2021.100087)
Supplement: Supplementary data 1 [file mmc1.docx]

| **Supplementary Material**  **Table S1**: Sociodemographic and clinical characteristics of mothers and their infants of those who remained in the cohort and those who missed follow-up   \|  \| **Vaccinated** \| \| \| \| \|  \| **Unvaccinated** \| \| \| \| \|  \| \| --- \| --- \| --- \| --- \| --- \| --- \| --- \| --- \| --- \| --- \| --- \| --- \| --- \| \|  \| Remained in the cohort (n=95) \| \|  \| Loss to follow-up (n=148) \| \|  \| Remained in the cohort (n=23) \| \|  \| Loss to follow-up (n=52) \| \|  \| \|  \| n \| % \|  \| n \| % \| p-value \| n \| % \|  \| n \| % \| p-value* \| \| **Maternal age (yrs)** \|  \| \|  \|  \|  \| 0.195 \|  \| \|  \|  \|  \| 0.788 \| \| <20 \| 4 \| 4.2 \|  \| 11 \| 7.4 \|  \| 2 \| 8.7 \|  \| 9 \| 17.3 \|  \| \| 20\|-30 \| 52 \| 54.7 \|  \| 80 \| 54.1 \|  \| 14 \| 60.9 \|  \| 29 \| 55.8 \|  \| \| 30\|-35 \| 17 \| 17.9 \|  \| 36 \| 24.3 \|  \| 3 \| 13.0 \|  \| 5 \| 9.6 \|  \| \| ≥35 \| 22 \| 23.2 \|  \| 21 \| 14.2 \|  \| 4 \| 17.4 \|  \| 9 \| 17.3 \|  \| \|  \|  \|  \|  \|  \|  \|  \|  \|  \|  \|  \|  \|  \| \| **Maternal race** \|  \|  \|  \|  \|  \| 0.046 \|  \|  \|  \|  \|  \| 0.802 \| \| White \| 26 \| 27.4 \|  \| 59 \| 39.9 \|  \| 10 \| 43.5 \|  \| 21 \| 40.4 \|  \| \| Non-white^#^ \| 69 \| 72.6 \|  \| 89 \| 60.1 \|  \| 13 \| 56.5 \|  \| 31 \| 59.6 \|  \| \|  \|  \|  \|  \|  \|  \|  \|  \|  \|  \|  \|  \|  \| \| **Mother educational level** \| \|  \|  \|  \|  \| 0.285 \|  \|  \|  \|  \|  \| 0.926 \| \| <8 years \| 37 \| 39.4 \|  \| 65 \| 46.4 \|  \| 10 \| 45.5 \|  \| 21 \| 46.7 \|  \| \| ≥ 8 years \| 57 \| 60.6 \|  \| 75 \| 53.6 \|  \| 12 \| 54.5 \|  \| 24 \| 53.3 \|  \| \|  \|  \|  \|  \|  \|  \|  \|  \|  \|  \|  \|  \|  \| \| **Household income per month** \| \|  \|  \|  \|  \| 0.438 \|  \|  \|  \|  \|  \| 0.074 \| \| R$ <1.576 ** \| 64 \| 67.4 \|  \| 87 \| 60.8 \|  \| 9 \| 40.9 \|  \| 35 \| 68.6 \|  \| \| R$ 1.576 - 3.151 \| 22 \| 23.2 \|  \| 44 \| 30.8 \|  \| 10 \| 45.5 \|  \| 11 \| 21.6 \|  \| \| ≥ R$ 3.152 \| 9 \| 9.5 \|  \| 12 \| 8.4 \|  \| 3 \| 13.6 \|  \| 5 \| 9.8 \|  \| \|  \|  \|  \|  \|  \|  \|  \|  \|  \|  \|  \|  \|  \| \| **Number of antenatal visits** \| \|  \|  \|  \|  \| 0.894 \|  \|  \|  \|  \|  \| 0.347 \| \| <7 \| 15 \| 15.8 \|  \| 24 \| 16.4 \|  \| 6 \| 26.1 \|  \| 19 \| 37.3 \|  \| \| ≥7 \| 80 \| 84.2 \|  \| 122 \| 83.6 \|  \| 17 \| 73.9 \|  \| 32 \| 62.7 \|  \| \|  \|  \|  \|  \|  \|  \|  \|  \|  \|  \|  \|  \|  \| \| **Gestation (wks)** \|  \|  \|  \|  \|  \| 0.703 \|  \|  \|  \|  \|  \| 0.731 \| \| 37\|-40 \| 55 \| 57.9 \|  \| 82 \| 55.4 \|  \| 15 \| 65.2 \|  \| 36 \| 69.2 \|  \| \| ≥40 \| 40 \| 42.1 \|  \| 66 \| 44.6 \|  \| 8 \| 34.8 \|  \| 16 \| 30.8 \|  \| \|  \|  \|  \|  \|  \|  \|  \|  \|  \|  \|  \|  \|  \| \| **Birthweight (g)** \|  \|  \|  \|  \|  \| 0.045 \|  \|  \|  \|  \|  \| 0.679 \| \| <3000 \| 9 \| 9.5 \|  \| 28 \| 18.9 \|  \| 5 \| 21.7 \|  \| 12 \| 23.1 \|  \| \| 3000\|-3500 \| 54 \| 56.8 \|  \| 63 \| 42.6 \|  \| 11 \| 47.8 \|  \| 29 \| 55.8 \|  \| \| ≥3500 \| 32 \| 33.7 \|  \| 57 \| 38.5 \|  \| 7 \| 30.4 \|  \| 11 \| 21.2 \|  \| \|  \|  \|  \|  \|  \|  \|  \|  \|  \|  \|  \|  \|  \| \| **Mode of delivery** \|  \|  \|  \|  \|  \| 0.457 \|  \|  \|  \|  \|  \| 0.569 \| \| Vaginal \| 48 \| 50.5 \|  \| 82 \| 55.4 \|  \| 13 \| 56.5 \|  \| 33 \| 63.5 \|  \| \| Cesarean section \| 47 \| 49.5 \|  \| 66 \| 44.6 \|  \| 10 \| 43.5 \|  \| 19 \| 36.5 \|  \| \| *Chi squared test,^#^=Non- white includes black, “pardo”(mixed race), Asian and indigenous;^**^=approximately US$277 \| \| \| \| \| \| \| \| \| \| \| \|  \| |
| --- | --- | --- | --- | --- | --- | --- | --- | --- | --- | --- | --- | --- | --- | --- | --- | --- | --- | --- | --- | --- | --- | --- | --- | --- | --- | --- | --- | --- | --- | --- | --- | --- | --- | --- | --- | --- | --- | --- | --- | --- | --- | --- | --- | --- | --- | --- | --- | --- | --- | --- | --- | --- | --- | --- | --- | --- | --- | --- | --- | --- | --- | --- | --- | --- | --- | --- | --- | --- | --- | --- | --- | --- | --- | --- | --- | --- | --- | --- | --- | --- | --- | --- | --- | --- | --- | --- | --- | --- | --- | --- | --- | --- | --- | --- | --- | --- | --- | --- | --- | --- | --- | --- | --- | --- | --- | --- | --- | --- | --- | --- | --- | --- | --- | --- | --- | --- | --- | --- | --- | --- | --- | --- | --- | --- | --- | --- | --- | --- | --- | --- | --- | --- | --- | --- | --- | --- | --- | --- | --- | --- | --- | --- | --- | --- | --- | --- | --- | --- | --- | --- | --- | --- | --- | --- | --- | --- | --- | --- | --- | --- | --- | --- | --- | --- | --- | --- | --- | --- | --- | --- | --- | --- | --- | --- | --- | --- | --- | --- | --- | --- | --- | --- | --- | --- | --- | --- | --- | --- | --- | --- | --- | --- | --- | --- | --- | --- | --- | --- | --- | --- | --- | --- | --- | --- | --- | --- | --- | --- | --- | --- | --- | --- | --- | --- | --- | --- | --- | --- | --- | --- | --- | --- | --- | --- | --- | --- | --- | --- | --- | --- | --- | --- | --- | --- | --- | --- | --- | --- | --- | --- | --- | --- | --- | --- | --- | --- | --- | --- | --- | --- | --- | --- | --- | --- | --- | --- | --- | --- | --- | --- | --- | --- | --- | --- | --- | --- | --- | --- | --- | --- | --- | --- | --- | --- | --- | --- | --- | --- | --- | --- | --- | --- | --- | --- | --- | --- | --- | --- | --- | --- | --- | --- | --- | --- | --- | --- | --- | --- | --- | --- | --- | --- | --- | --- | --- | --- | --- | --- | --- | --- | --- | --- | --- | --- | --- | --- | --- | --- | --- | --- | --- | --- | --- | --- | --- | --- | --- | --- | --- | --- | --- | --- | --- | --- | --- | --- | --- | --- | --- | --- | --- | --- | --- | --- | --- | --- | --- | --- | --- | --- | --- | --- | --- | --- | --- | --- | --- | --- | --- | --- | --- | --- | --- | --- | --- | --- | --- | --- | --- | --- | --- | --- | --- | --- | --- | --- | --- | --- | --- | --- | --- | --- | --- | --- | --- | --- | --- | --- | --- | --- | --- | --- | --- | --- | --- | --- | --- | --- | --- | --- | --- | --- | --- | --- | --- | --- | --- | --- | --- | --- | --- | --- | --- | --- | --- | --- | --- | --- | --- | --- | --- | --- | --- | --- | --- | --- | --- | --- | --- | --- | --- | --- | --- | --- | --- | --- | --- | --- | --- | --- | --- | --- | --- | --- | --- | --- | --- | --- | --- | --- | --- | --- | --- | --- | --- | --- | --- | --- | --- | --- | --- | --- | --- | --- | --- | --- | --- | --- | --- | --- | --- | --- | --- | --- | --- | --- | --- | --- | --- | --- | --- | --- | --- | --- | --- | --- | --- | --- | --- | --- | --- | --- | --- | --- | --- | --- | --- | --- | --- | --- | --- | --- | --- | --- | --- | --- | --- |

| **Table S2: Characteristics of the infants, Sao Paulo, 2015-2017** | | | | | | | | | |
| --- | --- | --- | --- | --- | --- | --- | --- | --- | --- |
|  | **Total** | |  | **Vaccinated** | |  | **Unvaccinated** | | **p*** |
|  | **N** | **%** |  | **N** | **%** |  | **N** | **%** |  |
| **Breastfeeding at 2 months**** |  |  |  |  |  |  |  |  | 0.745 |
| Yes | 163 | 95.3 |  | 130 | 95.6 |  | 33 | 94.3 |  |
| No | 8 | 4.7 |  | 6 | 4.4 |  | 2 | 5.7 |  |
|  |  |  |  |  |  |  |  |  |  |
| **Breastfeeding at 7 months**** |  |  |  |  |  |  |  |  | 0.319 |
| Yes | 80 | 64.5 |  | 66 | 66.7 |  | 14 | 56.0 |  |
| No | 44 | 35.5 |  | 33 | 33.3 |  | 11 | 44.0 |  |
|  |  |  |  |  |  |  |  |  |  |
| **Day care attendance at 7 months** |  |  |  |  |  |  |  |  | 0.064 |
| Yes | 16 | 12.9 |  | 10 | 10.1 |  | 6 | 24.0 |  |
| No | 108 | 87.1 |  | 89 | 89.9 |  | 19 | 76.0 |  |

***** Chi-Squared test

**
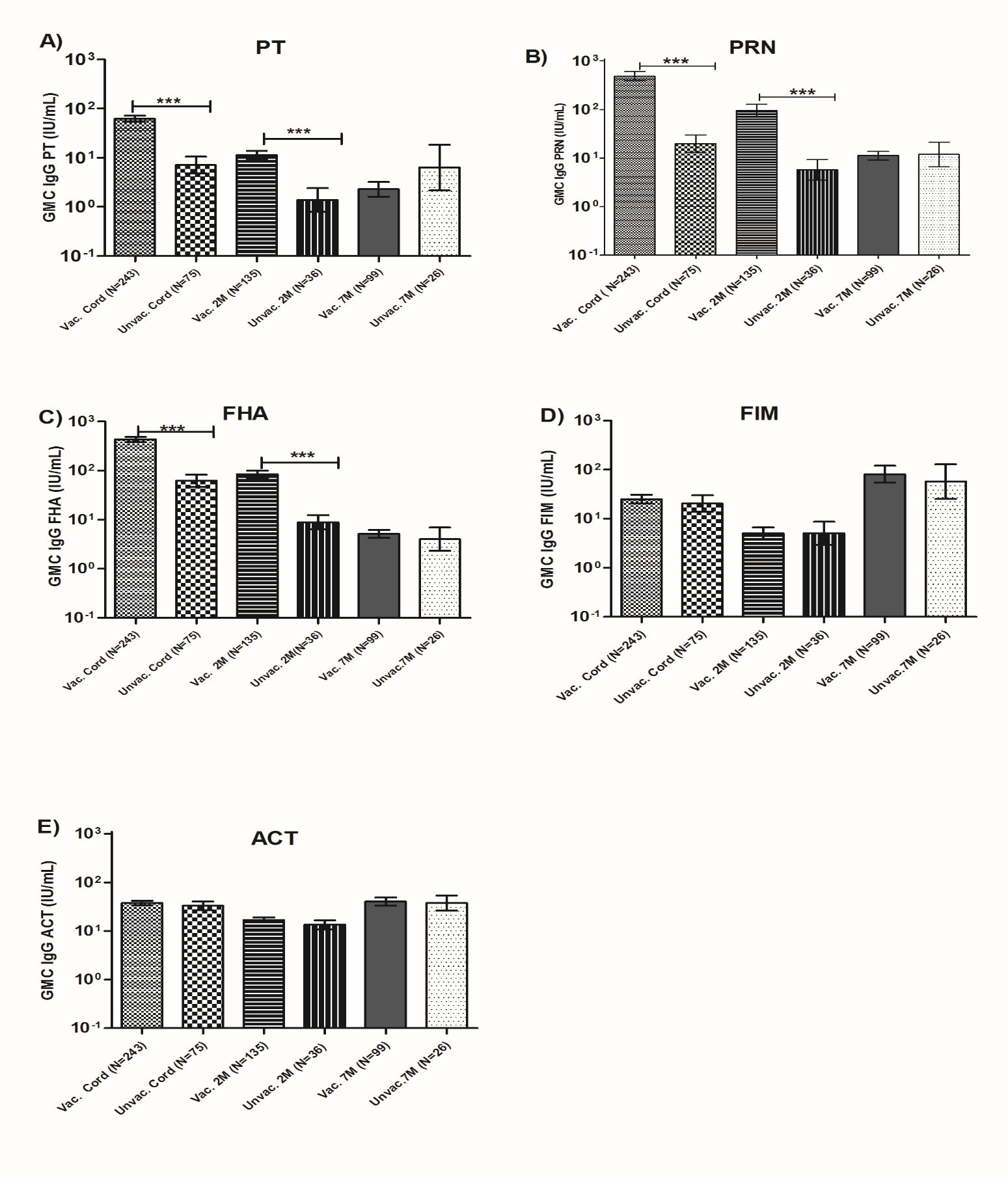
**

**Figure S1:** Geometric mean concentrations (GMCs) for IgG antibodies pertussis antigens PT (A), PRN (B), FHA (C), FIM (D) and ACT (E) in cord blood at delivery, infant plasma before primary vaccination (2M) and 1 month after the third vaccine dose (7M) for all infant samples collected. Statistical significance is indicated as ***= P<0.001. Mann Whitney test was used to analyze all variables except for FHA at 2 months, FHA, PRN and ACT at 7 months (Student’s t-test). The number of samples tested (N) for time points was indicated in graphs.

**Table S3:** **Influence of breastfeeding on infant IgG response at 2 months of age**

|  | Breastfeeding at 2 months of age | | | | | | |  |
| --- | --- | --- | --- | --- | --- | --- | --- | --- |
|  | Yes | | |  | No | | | p* |
|  | N | GMC | 95% CI |  | N | GMC | 95% CI |  |
| PT |  |  |  |  |  |  |  |  |
| Vaccinated | 130 | 11.70 | 9.55-14.33 |  | 6 | 5.13 | 1.20-21.91 | 0.085 |
| Unvaccinated** | 33 | 1.53 | 0.85-2.77 |  | 2 | 0.47 | 0.01-1503.43 | 0.227 |
|  |  |  |  |  |  |  |  |  |
| PRN |  |  |  |  |  |  |  |  |
| Vaccinated | 130 | 100.17 | 73.44-136.63 |  | 6 | 33.87 | 8.39-136.74 | 0.107 |
| Unvaccinated** | 33 | 5.42 | 3.23-9.10 |  | 2 | 19.76 | 0.01-611628.1 | 0.155 |
|  |  |  |  |  |  |  |  |  |
| FHA |  |  |  |  |  |  |  |  |
| Vaccinated | 130 | 88.08 | 73.25-105.91 |  | 6 | 29.59 | 8.64-101.26 | 0.029 |
| Unvaccinated** | 33 | 8.64 | 6.10-12.23 |  | 2 | 17.97 | 0.28-1150.49 | 0.200 |
|  |  |  |  |  |  |  |  |  |
| FIM |  |  |  |  |  |  |  |  |
| Vaccinated | 130 | 4.84 | 3.63-6.47 |  | 6 | 11.01 | 4.48-27.04 | 0.275 |
| Unvaccinated** | 33 | 4.47 | 2.53-7.89 |  | 2 | 22.93 | 0.0-5286854.00 | 0.135 |
|  |  |  |  |  |  |  |  |  |
| ACT |  |  |  |  |  |  |  |  |
| Vaccinated | 130 | 16.93 | 14.84-19.32 |  | 6 | 16.58 | 11.54-23.80 | 0.957 |
| Unvaccinated** | 33 | 13.34 | 10.57-16.85 |  | 2 | 15.23 | 1.47-157.27 | 0.669 |
|  |  |  |  |  |  |  |  |  |

* Mann-Whitney test; **=1 without information

**Table S4:** **Influence of breastfeeding on infant IgG response at 7 months of age**

|  | Breastfeeding at 7 months of age | | | | | | |  |
| --- | --- | --- | --- | --- | --- | --- | --- | --- |
|  | Yes | | |  | No | | | p* |
|  | N | GMC | 95% CI |  | N | GMC | 95% CI |  |
| PT |  |  |  |  |  |  |  |  |
| Vaccinated | 66 | 2.04 | 1.35-3.10 |  | 33 | 2.81 | 1.51-5.23 | 0.603 |
| Unvaccinated** | 14 | 6.60 | 1.44-30.11 |  | 11 | 5.43 | 0.77-37.89 | 0.956 |
|  |  |  |  |  |  |  |  |  |
| PRN |  |  |  |  |  |  |  |  |
| Vaccinated | 66 | 10.42 | 8.26-13.16 |  | 33 | 11.77 | 7.80-17.76 | 0.867 |
| Unvaccinated** | 14 | 16.10 | 7.38-35.11 |  | 11 | 10.08 | 3.84-26.47 | 0.511 |
|  |  |  |  |  |  |  |  |  |
| FHA |  |  |  |  |  |  |  |  |
| Vaccinated | 66 | 5.42 | 4.25-6.94 |  | 33 | 4.70 | 3.55-6.21 | 0.572 |
| Unvaccinated** | 14 | 5.31 | 2.55-11.06 |  | 11 | 3.00 | 1.10-8.20 | 0.661 |
|  |  |  |  |  |  |  |  |  |
| FIM |  |  |  |  |  |  |  |  |
| Vaccinated | 66 | 79.53 | 50.14-126.13 |  | 33 | 85.07 | 38.32-188.80 | 0.645 |
| Unvaccinated** | 14 | 66.29 | 21.98-199.94 |  | 11 | 45.95 | 9.68-217.96 | 0.443 |
|  |  |  |  |  |  |  |  |  |
| ACT |  |  |  |  |  |  |  |  |
| Vaccinated | 66 | 33.85 | 26.27-43.61 |  | 33 | 55.22 | 41.10-74.21 | 0.007 |
| Unvaccinated** | 14 | 51.08 | 31.69-82.33 |  | 11 | 29.43 | 16.86-51.37 | 0.188 |
|  |  |  |  |  |  |  |  |  |

***** Mann-Whitney test; ******=1 without information
